# 3-(4-Carb­oxy-5-carboxyl­ato-1*H*-imidazol-2-yl)pyridin-1-ium monohydrate

**DOI:** 10.1107/S1600536811002248

**Published:** 2011-01-26

**Authors:** Guang-Jun Liu, Guang-Wang Zhao, Li Li, Hong-Tao Gao

**Affiliations:** a Department of Chemisry and Chemical Engineering, Jining University, 273155 Qufu, Shandong, People’s Republic of China; b Shandong Lukang Pharmaceutical Group Co. Ltd, 272100 Jining, Shandong, People’s Republic of China

## Abstract

In the zwitterionic mol­ecule of the title compound, C_10_H_7_N_3_O_4_·H_2_O, one carboxyl group is deprotonated and the pyridine N atom is protonated. The pyridinium and imidazole rings form a dihedral angle of 5.23 (1)°. An intramolecular O—H⋯O hydrogen bond occurs. In the crystal, inter­molecular N—H⋯O, O—H⋯N and O—H⋯O hydrogen bonds link the zwitterions and water mol­ecules into sheets parallel to (102).

## Related literature

For the use of 4,5-imidazole­dicarb­oxy­lic acid in coordination chemistry and for related structures, see: Sun *et al.* (2006 ▶); Chen (2008 ▶); Liu *et al.* (2009 ▶). For the synthesis of the title compound, see: Lebedev *et al.* (2007 ▶). For bond-length data, see: Allen *et al.* (1987 ▶). 
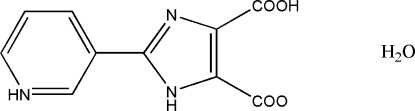


## Experimental

### 

#### Crystal data

C_10_H_7_N_3_O_4_·H_2_O
*M* *_r_* = 251.20
Monoclinic, 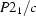

*a* = 3.7342 (18) Å
*b* = 16.354 (8) Å
*c* = 16.634 (8) Å
β = 97.019 (10)°
*V* = 1008.2 (8) Å^3^
*Z* = 4
Mo *K*α radiation
μ = 0.14 mm^−1^
*T* = 298 K
0.32 × 0.28 × 0.25 mm

#### Data collection

Bruker SMART APEX CCD area-detector diffractometer
Absorption correction: multi-scan (*SADABS*; Bruker, 2005 ▶) *T*_min_ = 0.958, *T*_max_ = 0.967
5038 measured reflections
1777 independent reflections
1314 reflections with *I* > 2σ(*I*)
*R*_int_ = 0.028

#### Refinement

*R*[*F* ^2^ > 2σ(*F* ^2^)] = 0.042
*wR*(*F* ^2^) = 0.119
*S* = 1.14
1777 reflections
179 parameters
H atoms treated by a mixture of independent and constrained refinement
Δρ_max_ = 0.21 e Å^−3^
Δρ_min_ = −0.18 e Å^−3^

### 

Data collection: *APEX2* (Bruker, 2005 ▶); cell refinement: *SAINT* (Bruker, 2005 ▶); data reduction: *SAINT*; program(s) used to solve structure: *SHELXTL* (Sheldrick, 2008 ▶); program(s) used to refine structure: *SHELXTL*; molecular graphics: *SHELXTL*; software used to prepare material for publication: *SHELXTL*.

## Supplementary Material

Crystal structure: contains datablocks global, I. DOI: 10.1107/S1600536811002248/cv5038sup1.cif

Structure factors: contains datablocks I. DOI: 10.1107/S1600536811002248/cv5038Isup2.hkl

Additional supplementary materials:  crystallographic information; 3D view; checkCIF report

## Figures and Tables

**Table 1:** Hydrogen-bond geometry (Å, °)

*D*—H⋯*A*	*D*—H	H⋯*A*	*D*⋯*A*	*D*—H⋯*A*
N1—H1⋯O4^i^	0.98 (2)	1.87 (2)	2.824 (3)	164.8 (16)
N3—H3⋯O1*W*^ii^	0.99 (3)	1.66 (3)	2.625 (3)	163 (2)
O1*W*—H1*A*⋯O3^iii^	0.94 (4)	1.84 (4)	2.782 (3)	176 (4)
O1*W*—H1*B*⋯O1	0.95 (4)	2.50 (4)	3.032 (3)	115 (3)
O1*W*—H1*B*⋯N2	0.95 (4)	1.90 (4)	2.839 (2)	166 (3)
O2—H2⋯O3	0.82	1.67	2.493 (2)	179

Symmetry codes: (i) 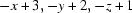; (ii) 
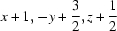
; (iii) 
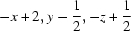
.

**Fig. 1.**
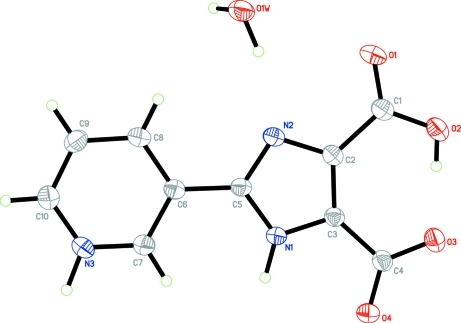
View of (I) showing the atomic numbering and 30% probabilty displacement ellipsoids.

**Fig. 2.**
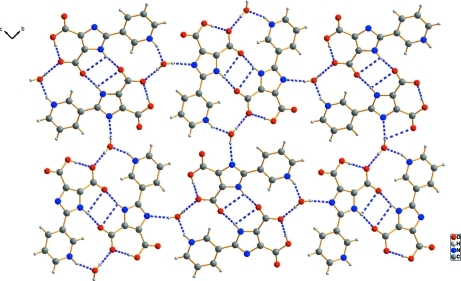
Portion of the crystal packing showing the sheet of hydrogen-bonded (dashed lines) molecules.

## supplementary crystallographic information

### Comment

Imidazole-4,5-dicarboxylic acid derivatives are recognized as efficient N,*O*-donors exhibiting diverse modes of coordination (Sun *et al.*, 2006; Chen, 2008; Liu *et al.*, 2009). In order to search for new derivatives of imidazole-4,5-dicarboxylic acid, the title compound (I) was synthesized and its crystal structure is reported here.

In (I)  (Fig. 1), all bond lengths and angles are normal (Allen *et al.*, 1987). The C4-containing carboxyl group is deprotonated and forms an intramolecular hydrogen bond with the neighboring C1-containing carboxyl group. The dihedral angle formed by imidazole ring and pyridinium ring is 5.23 (1) °. In the crystal structure, intermolecular N—H···O, O—H···N and O—H···O hydrogen bonds (Table 1) link the molecules into sheets parallel to (102) plane (Fig. 2). The short axis *a* of 3.7342 (18) Å suggests a presence of π-π interactions between the rings from the neighbouring sheets, which consolidate further the crystal packing.

### Experimental

The title compound was synthesized according to the method reported in the literature (Lebedev *et al.*, 2007). Yellow single crystals suitable for X-ray diffraction were obtained by slow evaporation of an acetonitrile solution of the compound.

### Refinement

H atoms bonded to N and O atoms were located in a difference Fourier map and refined isotropically. C-bound H atoms were placed in calculated positions with C—H = 0.93 Å, and refined as riding, with *U*_iso_(H) = 1.2*U*_iso_(C).

### Crystal data

**Table:**

C_10_H_7_N_3_O_4_·H_2_O	*F*(000) = 520
*M**_r_* = 251.20	*D*_x_ = 1.655 Mg m^−^^3^
Monoclinic, *P*2_1_/*c*	Mo *K*α radiation, λ = 0.71073 Å
Hall symbol: -P 2ybc	Cell parameters from 1155 reflections
*a* = 3.7342 (18) Å	θ = 2.5–21.9°
*b* = 16.354 (8) Å	µ = 0.14 mm^−^^1^
*c* = 16.634 (8) Å	*T* = 298 K
β = 97.019 (10)°	Block, yellow
*V* = 1008.2 (8) Å^3^	0.32 × 0.28 × 0.25 mm
*Z* = 4	

### Data collection

**Table:**

Bruker SMART APEX CCD area-detector diffractometer	1777 independent reflections
Radiation source: fine-focus sealed tube	1314 reflections with *I* > 2σ(*I*)
graphite	*R*_int_ = 0.028
φ and ω scans	θ_max_ = 25.1°, θ_min_ = 1.8°
Absorption correction: multi-scan (*SADABS*; Bruker, 2005)	*h* = −4→4
*T*_min_ = 0.958, *T*_max_ = 0.967	*k* = −18→19
5038 measured reflections	*l* = −19→15

### Refinement

**Table:**

Refinement on *F*^2^	Primary atom site location: structure-invariant direct methods
Least-squares matrix: full	Secondary atom site location: difference Fourier map
*R*[*F*^2^ > 2σ(*F*^2^)] = 0.042	Hydrogen site location: inferred from neighbouring sites
*wR*(*F*^2^) = 0.119	H atoms treated by a mixture of independent and constrained refinement
*S* = 1.14	*w* = 1/[σ^2^(*F*_o_^2^) + (0.0612*P*)^2^] where *P* = (*F*_o_^2^ + 2*F*_c_^2^)/3
1777 reflections	(Δ/σ)_max_ < 0.001
179 parameters	Δρ_max_ = 0.21 e Å^−^^3^
0 restraints	Δρ_min_ = −0.18 e Å^−^^3^

### Special details

**Table:**

Geometry. All e.s.d.'s (except the e.s.d. in the dihedral angle between two l.s. planes) are estimated using the full covariance matrix. The cell e.s.d.'s are taken into account individually in the estimation of e.s.d.'s in distances, angles and torsion angles; correlations between e.s.d.'s in cell parameters are only used when they are defined by crystal symmetry. An approximate (isotropic) treatment of cell e.s.d.'s is used for estimating e.s.d.'s involving l.s. planes.
Refinement. Refinement of *F*^2^ against ALL reflections. The weighted *R*-factor *wR* and goodness of fit *S* are based on *F*^2^, conventional *R*-factors *R* are based on *F*, with *F* set to zero for negative *F*^2^. The threshold expression of *F*^2^ > σ(*F*^2^) is used only for calculating *R*-factors(gt) *etc*. and is not relevant to the choice of reflections for refinement. *R*-factors based on *F*^2^ are statistically about twice as large as those based on *F*, and *R*- factors based on ALL data will be even larger.

### Fractional atomic coordinates and isotropic or equivalent isotropic displacement parameters (Å^2^)

**Table:**

	*x*	*y*	*z*	*U*_iso_*/*U*_eq_	
O1	0.5287 (5)	0.93482 (10)	0.16289 (9)	0.0556 (5)	
O2	0.7858 (5)	1.05235 (10)	0.19683 (9)	0.0573 (5)	
H2	0.9129	1.0721	0.2357	0.086*	
O3	1.1698 (5)	1.11399 (9)	0.31442 (9)	0.0576 (5)	
O4	1.4072 (5)	1.07667 (9)	0.43806 (9)	0.0525 (5)	
N1	1.1259 (5)	0.92199 (10)	0.41497 (10)	0.0348 (4)	
N2	0.7955 (5)	0.86368 (10)	0.31150 (9)	0.0356 (4)	
N3	1.0972 (6)	0.70365 (11)	0.55425 (11)	0.0455 (5)	
C1	0.7137 (6)	0.97627 (13)	0.21234 (13)	0.0415 (6)	
C2	0.8660 (6)	0.94325 (12)	0.29214 (11)	0.0347 (5)	
C3	1.0711 (5)	0.98027 (11)	0.35661 (11)	0.0335 (5)	
C4	1.2301 (6)	1.06322 (12)	0.37174 (12)	0.0391 (5)	
C5	0.9577 (6)	0.85300 (11)	0.38601 (11)	0.0329 (5)	
C6	0.9521 (6)	0.77649 (12)	0.43184 (12)	0.0338 (5)	
C7	1.1011 (6)	0.77284 (13)	0.51242 (12)	0.0396 (5)	
H7	1.2050	0.8194	0.5375	0.047*	
C8	0.7973 (6)	0.70592 (13)	0.39780 (13)	0.0424 (6)	
H8	0.6916	0.7062	0.3442	0.051*	
C9	0.7986 (7)	0.63518 (13)	0.44279 (13)	0.0466 (6)	
H9	0.6945	0.5877	0.4197	0.056*	
C10	0.9544 (7)	0.63514 (14)	0.52181 (13)	0.0478 (6)	
H10	0.9599	0.5875	0.5524	0.057*	
O1W	0.4229 (6)	0.75493 (12)	0.19730 (11)	0.0711 (6)	
H1	1.271 (5)	0.9320 (11)	0.4672 (12)	0.032 (5)*	
H3	1.211 (8)	0.7089 (16)	0.6111 (18)	0.080 (9)*	
H1B	0.556 (12)	0.797 (2)	0.228 (2)	0.143 (15)*	
H1A	0.570 (11)	0.709 (2)	0.193 (2)	0.122 (13)*	

### Atomic displacement parameters (Å^2^)

**Table:**

	*U*^11^	*U*^22^	*U*^33^	*U*^12^	*U*^13^	*U*^23^
O1	0.0640 (12)	0.0629 (10)	0.0351 (9)	−0.0017 (9)	−0.0135 (8)	−0.0030 (7)
O2	0.0700 (13)	0.0532 (10)	0.0441 (9)	−0.0053 (9)	−0.0114 (8)	0.0136 (7)
O3	0.0715 (13)	0.0442 (9)	0.0524 (10)	−0.0060 (8)	−0.0111 (9)	0.0120 (8)
O4	0.0650 (12)	0.0457 (9)	0.0419 (9)	−0.0067 (8)	−0.0127 (8)	−0.0030 (7)
N1	0.0393 (11)	0.0355 (9)	0.0286 (9)	−0.0006 (8)	0.0004 (8)	−0.0026 (7)
N2	0.0354 (11)	0.0405 (10)	0.0298 (9)	0.0014 (8)	−0.0001 (8)	−0.0026 (7)
N3	0.0547 (14)	0.0490 (11)	0.0312 (10)	0.0002 (9)	−0.0017 (9)	0.0036 (9)
C1	0.0408 (14)	0.0468 (13)	0.0363 (12)	0.0044 (11)	0.0021 (10)	−0.0003 (10)
C2	0.0340 (13)	0.0389 (11)	0.0313 (11)	0.0053 (9)	0.0048 (9)	0.0008 (9)
C3	0.0330 (12)	0.0355 (11)	0.0314 (10)	0.0047 (9)	0.0018 (9)	−0.0015 (9)
C4	0.0400 (14)	0.0394 (12)	0.0373 (12)	0.0049 (10)	0.0021 (10)	0.0019 (10)
C5	0.0331 (12)	0.0358 (11)	0.0292 (10)	0.0027 (9)	0.0013 (9)	−0.0044 (8)
C6	0.0296 (12)	0.0385 (11)	0.0331 (11)	0.0028 (9)	0.0027 (9)	−0.0015 (9)
C7	0.0409 (14)	0.0401 (12)	0.0366 (12)	−0.0007 (10)	0.0005 (10)	−0.0009 (9)
C8	0.0451 (15)	0.0466 (12)	0.0337 (12)	−0.0034 (10)	−0.0027 (10)	−0.0026 (10)
C9	0.0492 (15)	0.0401 (12)	0.0501 (14)	−0.0070 (11)	0.0045 (11)	−0.0059 (10)
C10	0.0524 (16)	0.0460 (13)	0.0447 (14)	−0.0051 (11)	0.0044 (12)	0.0052 (10)
O1W	0.1032 (17)	0.0470 (11)	0.0534 (11)	0.0091 (11)	−0.0304 (11)	−0.0099 (8)

### Geometric parameters (Å, °)

**Table:**

O1—C1	1.214 (2)	C2—C3	1.379 (3)
O2—C1	1.305 (3)	C3—C4	1.490 (3)
O2—H2	0.8201	C5—C6	1.467 (3)
O3—C4	1.264 (2)	C6—C8	1.381 (3)
O4—C4	1.235 (2)	C6—C7	1.388 (3)
N1—C5	1.351 (2)	C7—H7	0.9300
N1—C3	1.358 (3)	C8—C9	1.377 (3)
N1—H1	0.98 (2)	C8—H8	0.9300
N2—C5	1.323 (2)	C9—C10	1.371 (3)
N2—C2	1.374 (3)	C9—H9	0.9300
N3—C10	1.327 (3)	C10—H10	0.9300
N3—C7	1.329 (3)	O1W—H1B	0.95 (4)
N3—H3	0.99 (3)	O1W—H1A	0.94 (4)
C1—C2	1.481 (3)		
			
C1—O2—H2	109.5	N2—C5—N1	111.31 (17)
C5—N1—C3	107.96 (17)	N2—C5—C6	124.45 (18)
C5—N1—H1	129.6 (10)	N1—C5—C6	124.24 (17)
C3—N1—H1	122.4 (11)	C8—C6—C7	117.27 (19)
C5—N2—C2	105.42 (16)	C8—C6—C5	122.13 (18)
C10—N3—C7	122.4 (2)	C7—C6—C5	120.60 (18)
C10—N3—H3	124.4 (16)	N3—C7—C6	120.88 (19)
C7—N3—H3	113.2 (16)	N3—C7—H7	119.6
O1—C1—O2	120.8 (2)	C6—C7—H7	119.6
O1—C1—C2	121.9 (2)	C9—C8—C6	120.4 (2)
O2—C1—C2	117.32 (19)	C9—C8—H8	119.8
N2—C2—C3	109.74 (17)	C6—C8—H8	119.8
N2—C2—C1	119.47 (18)	C10—C9—C8	119.6 (2)
C3—C2—C1	130.76 (19)	C10—C9—H9	120.2
N1—C3—C2	105.58 (17)	C8—C9—H9	120.2
N1—C3—C4	119.74 (17)	N3—C10—C9	119.5 (2)
C2—C3—C4	134.67 (18)	N3—C10—H10	120.3
O4—C4—O3	125.7 (2)	C9—C10—H10	120.3
O4—C4—C3	118.17 (18)	H1B—O1W—H1A	110 (4)
O3—C4—C3	116.11 (18)		
			
C5—N2—C2—C3	−0.5 (2)	C2—N2—C5—N1	0.3 (2)
C5—N2—C2—C1	−178.48 (19)	C2—N2—C5—C6	179.57 (19)
O1—C1—C2—N2	−0.4 (3)	C3—N1—C5—N2	−0.1 (2)
O2—C1—C2—N2	179.53 (19)	C3—N1—C5—C6	−179.32 (19)
O1—C1—C2—C3	−178.0 (2)	N2—C5—C6—C8	5.3 (3)
O2—C1—C2—C3	2.0 (4)	N1—C5—C6—C8	−175.5 (2)
C5—N1—C3—C2	−0.2 (2)	N2—C5—C6—C7	−174.0 (2)
C5—N1—C3—C4	−179.47 (18)	N1—C5—C6—C7	5.2 (3)
N2—C2—C3—N1	0.4 (2)	C10—N3—C7—C6	0.3 (3)
C1—C2—C3—N1	178.2 (2)	C8—C6—C7—N3	0.8 (3)
N2—C2—C3—C4	179.5 (2)	C5—C6—C7—N3	−179.91 (19)
C1—C2—C3—C4	−2.8 (4)	C7—C6—C8—C9	−0.9 (3)
N1—C3—C4—O4	−0.5 (3)	C5—C6—C8—C9	179.8 (2)
C2—C3—C4—O4	−179.5 (2)	C6—C8—C9—C10	0.1 (3)
N1—C3—C4—O3	179.49 (19)	C7—N3—C10—C9	−1.2 (4)
C2—C3—C4—O3	0.5 (4)	C8—C9—C10—N3	1.0 (4)

### Hydrogen-bond geometry (Å, °)

**Table:**

*D*—H···*A*	*D*—H	H···*A*	*D*···*A*	*D*—H···*A*
N1—H1···O4^i^	0.98 (2)	1.87 (2)	2.824 (3)	164.8 (16)
N3—H3···O1W^ii^	0.99 (3)	1.66 (3)	2.625 (3)	163 (2)
O1W—H1A···O3^iii^	0.94 (4)	1.84 (4)	2.782 (3)	176 (4)
O1W—H1B···O1	0.95 (4)	2.50 (4)	3.032 (3)	115 (3)
O1W—H1B···N2	0.95 (4)	1.90 (4)	2.839 (2)	166 (3)
O2—H2···O3	0.82	1.67	2.493 (2)	179

Symmetry codes: (i) −*x*+3, −*y*+2, −*z*+1; (ii) *x*+1, −*y*+3/2, *z*+1/2; (iii) −*x*+2, *y*−1/2, −*z*+1/2.
